# Surgical procedures in the treatment of 784 infected THAs reported to the Norwegian Arthroplasty Register: Best survival with 2-stage exchange revision, but also good results with debridement and retention of the fixed implant

**DOI:** 10.3109/17453674.2011.623572

**Published:** 2011-11-24

**Authors:** Lars B Engesæter, Håvard Dale, Jan C Schrama, Geir Hallan, Stein Atle Lie

**Affiliations:** ^1^The Norwegian Arthroplasty Register, Department of Orthopedic Surgery, Haukeland University Hospital; ^2^Institute of Surgical Sciences, University of Bergen, Bergen, Norway

## Abstract

**Background and purpose:**

Controversies still exist regarding the best surgical procedure in the treatment of periprosthetic infection after total hip arthroplasty (THA). Based on data in the Norwegian Arthroplasty Register (NAR), we have compared the risk of re-revision after 4 different surgical procedures: 2-stage with exchange of the whole prosthesis, 1-stage with exchange of the whole prosthesis, major partial 1-stage with exchange of stem or cup, and minor partial 1-stage with exchange of femoral head and/or acetabular liner.

**Methods:**

Between 1987 and 2009, 124,759 primary THAs were reported to the NAR, of which 906 (0.7%) were revised due to infection. Included in this study were the 784 revisions that had been performed by 1 of the 4 different surgical procedures. Cox-estimated survival and relative revision risks are presented with adjustment for differences among groups regarding gender, type of fixation, type of prosthesis, and age at revision.

**Results:**

2-stage procedures were used in 283 revisions (36%), 1-stage in 192 revisions (25%), major partial in 129 revisions (17%), and minor partial in 180 revisions (23%). 2-year Kaplan-Meier survival for all revisions was 83%; it was 92% for those re-revised by 2-stage exchange procedure, 88% for those re-revised by 1-stage exchange procedure, 66% for those re-revised by major partial exchange procedure, and it was 76% for those re-revised by minor partial exchange. Compared to the 2-stage procedure and with any reason for revision as endpoint (180 re-revisions), the risk of re-revision increased 1.4 times for 1-stage (p = 0.2), 4.1 times for major partial exchange (p < 0.001), and 1.5 times for minor partial exchange (p = 0.1). With infection as the endpoint (108 re-revisions), the risk of re-revision increased 2.0 times for 1-stage exchange (p = 0.04), 6.0 times for major partial exchange (p < 0.001), and 2.3 times for minor partial exchange (p = 0.02). Similar results were found when the analyses were restricted to the period 2002–2009.

**Interpretation:**

In the Norwegian Arthroplasty Register, the survival after revision of infected primary THA with 2-stage implant exchange was slightly superior to that for 1-stage exchange of the whole prosthesis. This result is noteworthy, since 2-stage procedures are often used with the most severe infections. However, debridement with exchange of head and/or liner but with retention of the fixed implant (minor revision) meant that there was a 76% chance of not being re-revised within 2 years.

The risk of periprosthetic infection after total hip arthroplasty (THA) has decreased from 5–10% in the late 1960s to around 1% today (Lidgren 2001, Lidgren et al. 2003, Zimmerli and Ochsner 2003, Dale et al. 2009, Walenkamp 2009, Ong et al. 2009, Urquhart et al. 2010). In the last few years, however, some reports have indicated that there is an increasing incidence of revisions for infected THA (Kurtz et al. 2008, Dale et al. 2009, Pedersen et al. 2010).

The optimum treatment of deep infection remains controversial (Toms et al. 2006). 2-stage exchange requires a minimum of 2 surgical procedures and a substantial period of reduced mobility. Removal of a well-fixed cemented prosthesis may also result in degradation of the bone stock and perioperative fracture (Langlais 2003, Ong et al. 2009, Bejon et al. 2010). A procedure with debridement, antibiotics, and implant retention (DAIR) with or without exchange of removable parts (i.e. liner and/or head), which is technically less demanding, could therefore be an attractive option for treatment of early deep infections, especially in elderly or frail patients (Giulieri et al. 2004, Trebse et al. 2005, Toms et al. 2006, Byren et al. 2009).

In this paper, based on the data in the Norwegian Arthroplasty Register (NAR), we compared the risk of re-revision after the first surgical revision for deep infection of a primary THA by one of the following 4 surgical procedures: 2-stage exchange of the whole prosthesis, 1-stage exchange of the whole prosthesis, major partial 1-stage with exchange of stem or acetabulum, and minor partial 1-stage with exchange of acetabular liner and/or femoral head (Trampuz and Zimmerli 2006).

## Patients and methods

The Norwegian Arthroplasty Register is a nationwide registry that was established in 1987. Each THA, primary or revision, performed in Norway is reported individually by the surgeon by completing a standard form (Havelin et al. 2000). Using the unique identification number assigned to each resident of Norway, the information from the primary THA is linked to any subsequent revisions in the registry. Revision is defined as surgical removal or exchange of the whole implant or part of it. Information in the form includes the identity of the patient, the date of the operation, the indications for surgery, the type of prosthesis (modular or monoblock), the type of fixation (uncemented, cemented with antibiotic-loaded cement, or cemented with cement without antibiotic), and the duration of the operation.

From the start of the Register in September 1987 to the end of December 2009, 124,759 primary THAs were reported. Included in the present study were first revisions of THAs performed due to deep infection. The decision as to the diagnosis of infection was made by the reporting orthopedic surgeon immediately postoperatively and therefore before the results of the peroperative culture were known. The postoperative systemic antibiotic treatment of the infection was not reported to the registry. The risk of re-revision of these revisions was compared for the following 4 types of surgical procedures for deep infection: 2-stage exchange revision in which the whole prosthesis was exchanged in a 2-stage procedure (following the removal of the infected implant, a new implant was inserted in a second stage), 1-stage exchange revision in which the whole prosthesis was exchanged in 1 operation, major partial 1-stage revision including exchange of the stem or the cup, or minor partial 1-stage revision with exchange of only the femoral head and/or the acetabular liner. Due to some changes in the strategy for surgical treatment of infected THAs in Norway in recent years, separate analyses were conducted for revisions performed during the latter part of the study period (2002–2009).

In addition, separate analyses were performed for revisions performed with time intervals from the primary THA to the revision of the infected implant of less than 3 weeks, of 3–12 weeks, and of more than 12 weeks.

### Statistics

Survival analysis was performed using the Kaplan-Meier and Cox regression methods. Patients who had died or emigrated during follow-up were identified from files provided by Statistics Norway, and the follow-up time for prostheses in these patients was censored at the date of death or emigration. A Cox multiple regression model was used to study relative revision risks (failure-rate ratios) among the 4 types of revision procedures, with adjustments for possible influence of gender, type of fixation (uncemented or cemented with or without antibiotic), type of prosthesis (monoblock, modular), and age of the patient at revision. Estimates from the Cox analyses with the 4 types of procedures as strata factors were used to construct adjusted survival curves. Assessments of proportionality in the Cox models were performed using log minus log of the adjusted survival curves, and the proportionality assumptions were fulfilled. SPSS software version 17.0 was used for the analyses.

## Results

**Figure 1.**
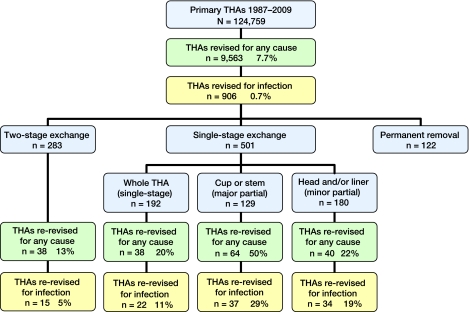
Flow chart showing the entire THA cohort and the different subgroups of surgical revision procedures (number and percentage) for revisions performed for any cause and due to infection.

Of the 124,759 primary THAs in the NAR, 9,563 (7.7%) were revised with removal or exchange of the whole prosthesis or parts of it. Of these, 906 were revised due to infection, i.e. 0.7% of all primary THAs or 9.5% of all first revisions (Figure 1). 122 revisions were permanent removals of the implant without later insertion of a new prosthesis (Girdlestone procedures). These were excluded in the subsequent analyses. The remaining 784 infected THAs were revised in the following ways: 2-stage exchange of the whole prosthesis (n = 283), 1-stage exchange of the whole prosthesis (n = 192), major partial 1-stage exchange of the stem or the acetabulum (n = 129), or minor partial 1-stage with exchange of the femoral head and/or the acetabular liner only (n = 180) (Table 1 and Figure 1).

**Table 1.** Descriptive data on 2-stage revision, 1-stage revision, major partial 1-stage exchange (i.e. exchange of stem or cup), and minor partial 1-stage exchange (i.e. exchange of head and/or liner) for infected THAs in the period 1987–2009

Type of revision	No. of revisions (%)	Mean age at revision (years) **^a^**	Females %	Mean duration of operation (min) **^a^**	Mean interval: operation to revision (years) **^a^**
2-stage	283 (36%)	71 (9; 34–88)	52%	320 (91; 155–780) **^b^**	2.9 (3.0; 0.1–16)
1-stage	192 (25%)	72 (9; 42–90)	45%	167 (59; 65–380) p < 0.001	2.9 (3.0; 0.0–15) p = 1.00
Major partial exchange	129 (17%)	70 (11; 31–89)	51%	139 (49; 35–300) p < 0.001	3.1 (3.4; 0.0–19) p = 1.00
Minor partial exchange	180 (23%)	72 (11; 38–92)	54%	94 (33; 35–320) p < 0.001	0.7 (2.0; 0.0–13) p < 0.001
Total	784 (100%)	71 (10; 31–92)	51%	202 (115; 35–780 )	2.4 (3.0; 0.0–19)

**^a^** Values are mean (SD; min–max)

**^b^** The duration of operation for 2-stage revision includes first stage with removal of the implant (171 (62; 45–445)) and second stage with re-implantation (149 (48; 35–450)).

Of these 784 revisions, 180 (23%) were re-revised: 13% of them with a 2-stage procedure, 20% with a 1-stage procedure, 50% with major partial, and 22% with minor partial (Figure 1). 2-year Kaplan-Meier survival for all revisions was 83%; for 2-stage exchange it was 92%, for 1-stage exchange it was 88%, for major partial exchange it was 66%, and for minor partial exchange it was 76% (Table 2).

**Table 2.** Results of the first revision for infected THA in the period 1987–2009 with any reason for revision and with infection as endpoints in the analyses for 2-stage revision, 1-stage revision, major partial 1-stage exchange (i.e. exchange of stem or cup), and minor partial 1-stage exchange (i.e. exchange of head and/or liner). Number of THAs, number of re-revisions (with complete dataset in the Cox analyses), Kaplan-Meier (KM) 2-year revision percentages, Cox relative revision risk (RR) (with 2-stage revision as reference), 95% confidence interval for RR, and p-value

Endpoint in the analyses	THA (%)	Re-revisions	2-year KM survival	RR **^a^**	95% CI	p-value
Any reason for revision:	784 (100%)	180 (23%)	83%			
2-stage revision	283 (36%)	38 (13%)	92%	1	–	–
1-stage revision	192 (25%)	38 (20%)	88%	1.4	0.9–2.1	0.2
Major partial exchange	129 (17%)	64 (50%)	66%	4.1	2.8–6.2	< 0.001
Minor partial exchange	180 (23%)	40 (22%)	76%	1.5	0.9–2.5	0.1
Infection:	784 (100%)	108 (14%)	88%			
2-stage revision	283 (36%)	15 (5%)	96%	1	–	–
1-stage revision	192 (25%)	22 (11%)	92%	2.0	1.1–3.9	0.04
Major partial exchange	129 (17%)	37 (29%)	74%	6.0	3.3–11.0	< 0.001
Minor partial exchange	180 (23%)	34 (19%)	80%	2.3	1.2–4.5	0.02

**^a^** Adjusted in the Cox model for sex, type of fixation, type of prosthesis, and age at revision.

**Figure 2.**
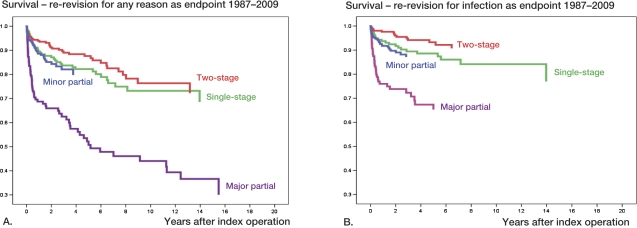
Cox-adjusted survival curves for first revision due to infected THA for the whole period 1987–2009 with any reason for re-revision (panel A) and infection (panel B) as endpoints in the analyses for 2-stage revision, 1-stage revision, major partial 1-stage exchange (i.e. exchange of stem or cup), and minor partial 1-stage exchange (i.e. exchange of head and/or liner).

With any reason for re-revision as endpoint in the Cox analyses, and compared to the 2-stage procedure, the risk of re-revision was 1.4 times higher for 1-stage exchange (p = 0.2), 4.1 times higher for major partial exchange (p < 0.001), and 1.5 times higher for minor partial exchange (p = 0.1) (Figure 2A and Table 2).

Of the 180 re-revisions, 108 (60%) were performed due to infection. For the 2-stage procedures, the re-revisions were performed due to infection in 39% of the cases (15 of 38); for the 1-stage procedures they were performed due to infection in 58% of cases (22 of 38); for major partial exchange they were performed due to infection in 58% of cases (37 of 64); and for minor partial exchange they were performed due to infection in 85% of cases (34 of 40).

With infection as endpoint in the Cox analyses, compared to the 2-stage procedure, the risk of re-revision was 2.0 times higher for 1-stage exchange (p = 0.04), 6.0 times higher for major partial exchange (p < 0.001), and 2.3 times higher for minor partial exchange (p = 0.02) (Figure 2B and Table 2).

**Figure 3.**
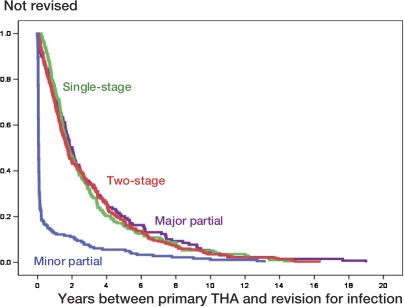
Interval between the primary THA and the first revision performed in the period 1987–2009, for 2-stage revision, 1-stage revision, and major and minor partial exchange.

The time interval between the primary THA and the first revision due to infection was shorter for the minor partial exchange than for the other procedures (Figure 3 and Table 1).

### The period 2002–2009

**Figure 4.**
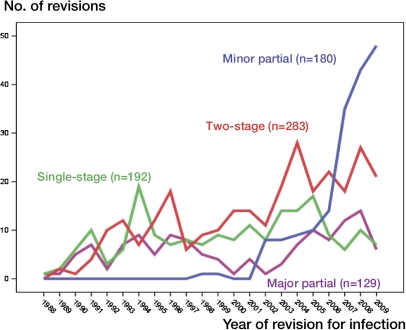
Number of first revisions per year for THAs carried out between 1987 and 2009 with the 2-stage procedure (283 revisions), 1-stage procedure (192 revisions), major partial 1-stage exchange (i.e. exchange of stem or cup) (129 revisions), and minor partial 1-stage exchange (i.e. exchange of the liner or head) (180 revisions).

In recent years, there has been a change in strategy for surgical revision of infected THAs, with a marked increase in minor partial exchange from 2002, and this procedure has now become the most common one for revision of infected THAs (Figure 4). Thus, subanalyses restricted to the period 2002–2009 were conducted and descriptive data are given in Table 3.

**Table 3.** Descriptive data on 2-stage revision, 1-stage revision, major partial 1-stage exchange (i.e. exchange of stem or cup), and minor partial 1-stage exchange (i.e. exchange of head and/liner) for infected THAs in the period 2002–2009

Type of revision	No. of revisions (%)	Mean age at revision (years) **^a^**	Females %	Mean duration of operation (min) **^a^**	Mean interval: operation to revision (years) **^a^**
2-stage revision	164 (34%)	71 (10; 39–87)	49%	330 (94; 175–780) **^b^**	3.2 (3.5; 0.1–16.3)
1-stage revision	86 (18%)	73 (10; 42–89)	43%	168 (63; 70–380) p < 0.001	3.7 (3.7; 0.2–14.9) p = 1.00
Major partial exchange	61 (13%)	71 (13; 31–89)	48%	139 (48; 40–265) p < 0.001	3.7 (4.3; 0.0–19.0) p = 1.00
Minor partial exchange	178 (36%)	71 (11; 38–89)	53%	95 (34; 35–320) p < 0.001	0.7 (2.0;0.0–13.1) p < 0.001
Total	489 (100%)	71 (10; 38–89)	50%	197 (121; 35–780 )	2.5 (3.5; 0.0–19.0)

**^a^** Values are mean (SD; min–max)

**^b^** The duration of operation for 2-stage revision includes first stage with removal of the implant (179 (64; 45–445)) and second stage with re-implantation (151 (52; 70–450)).

Of the 489 first revisions due to infection in 2002–2009, 100 (20%) were reported to be re-revised (for any reason) during this shorter follow-up until the end of 2009, i.e. up to 8 years. For those treated with the 2-stage procedure, 9% were re-revised; for the 1-stage procedure, 13% were re-revised; for the major partial procedure, 56% were re-revised; and for the minor partial procedure, 23% were re-revised. 2-year Kaplan-Meier survival for revisions performed in the period 2002–2009 was about the same as for the whole period (1987–2009), but there was inferior survival for major partial exchange (Table 4).

**Table 4.** Results of the first revision for infected THA in the period 2002–2009 with any reason for re-revision and with infection as endpoints in the analyses for 2-stage revision, 1-stage revision, major partial 1-stage exchange (i.e. exchange of stem or cup), and minor partial 1-stage exchange (i.e. exchange of head and/or liner). Number of THAs, number of re-revisions (with complete dataset in the Cox analyses), Kaplan-Meier (KM) 2-year revision percentages, Cox relative revision risk (RR) (with 2-stage revision as reference), 95% confidence interval for RR, and p-value

Endpoint in the analyses	THA (%)	Re-revisions	2-year KM survival	RR **^a^**	95% CI	p-value
Any reason for revision:	489 (100%)	100 (23%)	83%			
2-stage revision	164 (34%)	15 (9%)	94%	1	–	–
1-stage revision	86 (18%)	11 (13%)	87%	1.3	0.6–2.8	0.5
Major partial exchange	61 (12%)	34 (56%)	57%	7.4	4.0–13.8	< 0.001
Minor partial exchange	178 (36%)	40 (22%)	78%	1.9	1.0–3.8	0.05
Infection:	489 (100%)	77 (16%)	86%			
2-stage revision	164 (34%)	9 (5%)	98%	1	–	–
1-stage revision	86 (18%)	8 (9%)	89%	1.6	0.6–4.2	0.4
Major partial exchange	61 (12%)	26 (43%)	63%	9.0	4.2–19.5	< 0.001
Minor partial exchange	178 (36%)	34 (19%)	80%	2.5	1.1–5.8	0.03

**^a^** Adjusted in the Cox model for sex, type of fixation, type of prosthesis, and age at revision.

**Figure 5.**
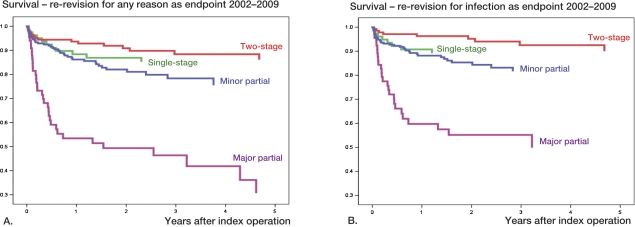
Cox-adjusted survival curves for first revision due to infected THA for 2002–2009 with any reason for re-revision (panel A) and infection (panel B) as endpoints in the analyses for 2-stage revision, 1-stage revision, major partial 1-stage exchange (i.e. exchange of stem or cup), and minor partial 1-stage exchange (i.e. exchange of head and/or liner) .

With any reason for re-revision as endpoint, for the period 2002–2009 and compared to 2-stage exchange revision, the risk of re-revision was 1.3 times higher for 1-stage exchange (p = 0.5), 7.4 times higher for major partial exchange (p < 0.001), and 1.9 times higher for minor partial exchange (p = 0.05) (Table 4 and Figure 5A).

For the period 2002–2009, 77 of the 100 re-revisions were performed due to infection: 2-stage re-revisions were performed in 9 hips, 1-stage in 8, major partial in 26, and minor partial in 34 hips. With infection as endpoint for re-revision, the risk of re-revision for 1-stage exchange was 1.6 times higher (p = 0.4), it was 9.0 times higher for major partial exchange (p < 0.001), and 2.5 times higher for minor partial exchange (p = 0.03) (Table 4 and Figure 5B).

### Revisions with uncemented and cemented THAs

For the whole period 1987–2009, 201 revisions were performed with uncemented implants both in the femur and in the acetabulum and 252 revisions were performed with antibiotic-loaded cement in both components. Compared to uncemented implants and with any reason for re-revision as endpoint in the Cox analyses, we found no difference in the risk of re-revision for those revised with cemented implants (RR = 1.3 (0.8–2.0), p = 0.3). We obtained similar results using infection as endpoint (RR = 0.98 (0.5–1.9), p = 1.0).

During the period 2002–2009, 107 revisions were performed with uncemented prostheses and 87 with antibiotic-loaded cement. With any reason for re-revision as endpoint in the Cox analyses, the procedures performed with antibiotic-loaded cement had a risk ratio for re-revision of 1.1 (0.5–2.5) compared to uncemented prostheses (p = 0.8). With infection as endpoint, the risk ratio for re-revision was 1.1 (0.4–3.1) (p = 0.8).

### Time interval between primary THA and revision

Recently, there has been interest in the time interval between the primary THA and the revision due to infection. We therefore conducted separate analyses for the period 2002–2009 for revisions performed less than 3 weeks after the primary THA (73 revisions, 15%), 3–12 weeks after the primary THA (101 revisions, 21%), and more than 12 weeks after the primary THA (315 revisions, 64%). In the Cox analysis, either with any reason for revision or with infection as endpoint, no effect of the time interval between the primary operation and revision on the outcome of the revision was found, either for all procedures as a group or when the analyses were performed stratified for 2-stage, 1-stage, major, or minor partial revision procedures.

## Discussion

Revision rate due to infection after primary THA of 0.7% and 2-year Kaplan-Meier survival of 92% for 2-stage exchange procedure and of 88% for 1-stage exchange procedure is acceptable for a whole nation. 2-year Kaplan-Meier survival of 76% for the far less extensive surgical procedure, minor partial revision, was only slightly inferior. This simpler procedure—both for the patient and for the health system, with only a slightly increased risk of re-revision—might therefore be an attractive low-morbidity option for periprosthetic hip infection.

In this national surveillance study, 9.5% of all first revisions were performed due to infection. In a large, nationally representative population-based study of 51,345 revision THA procedures in the USA, 15% were done because of infection (Bozic et al. 2009).

Our investigation was a national, prospective observational study and the treatment of the reported infected THAs was not randomized with respect to the 4 different surgical procedures. Thus, the treatments are based on the surgeons' and the hospitals' experience, knowledge, and strategies. The 2-stage procedure tended to be used when there were more severe infections, and the debridement with retention of the fixed implant (minor revision) tended to be used in the less severe cases.

Our finding of 92% survival for the 2-stage procedure is in accordance with what is written in the literature (Stockley et al. 2008, Biring et al. 2009). Medical and surgical treatments are chosen individually by the treating surgeons on the basis of the different clinical settings. In the clinical setting, however, direct comparisons of these different surgical procedures are not appropriate since they tend to be used for different clinical indications.

In Norway, a 2-stage surgical procedure is usually chosen for the most severe infections. The best results for this procedure are therefore convincing. However, 2-stage surgery requires a substantial period of reduced mobility for the patient, and the total duration of surgery (removal and re-insertion of the implant) is approximately twice that of the other procedures (Tables 1 and 3). The removal of a soundly fixed prosthesis may also result in degradation of the bone stock and perioperative fracture (Stockley et al. 2008, Biring et al. 2009, Fink 2009).

In recent years, the simpler procedure, with aggressive surgical debridement and exchange of modular parts of the prosthesis, has become more popular in Norway (Figure 2) which is in accordance with experience in the literature (Giulieri et al. 2004, Trebse et al. 2005, Toms et al. 2006, Marculescu et al. 2006). This procedure is recommended in patients with a short history of infection (2–5 weeks), intact soft tissues (no fistulae), and bacteria that respond to biofilm antibiotics (Zimmerli et al. 2004). The reported results of such early debridement and implant retention vary from 30% eradication to 80% eradication of the infection (Azzam et al. 2010). In the present study, 76% of patients treated with debridement and retention did not have any further reported revisions to their hips during the 2 years of follow-up. Since we do not have follow-up data other than for revision surgery, the 76% does not represent the eradication rate. Some patients may still have had their infection but had not, for some reason, been re-revised.

During recent years in Norway, there has been an increase in the reported number of revisions of infected THAs (Dale et al. 2009, Walenkamp 2009). Since revisions of monoblock prostheses—without removing or exchanging part of the implant—are not reported to the registry, only revisions with removal or exchange of the whole or part of the prosthesis are covered by the reporting requirements. With modular prostheses, which nowadays represent more than 90% of the femoral stems, easily removable parts such as head and liner are exchanged and the operation is then reported to the registry as a revision. In the period under study, from 1987–2009, practically all uncemented THAs performed in Norway were modular (Norwegian Arthroplasty Register, Annual Report 2009). Of the cemented prostheses, about 20% were modular at the beginning of the period, but this proportion steadily increased to 90% at the end of the period. Partial exchange revision has thus been more feasible, which may have contributed to the dramatic increase in partial 1-stage exchange revision since 2000, as shown in Figure 2. This increase in minor partial 1-stage exchange revision could then partially explain the reported increase in revision for infection in Norway (Dale et al. 2009, Walenkamp 2009).

Major partial exchange, i.e. exchange of the stem or cup, takes about the same operating time as 1-stage exchange of the whole implant, but with inferior results. Thus, our findings do not support exchange of just the stem or cup in revision of an infected THA.

60% of all re-revisions were performed because of infections, which is in accordance with what has been published (Jafari et al. 2010). For those revised with 2-stage revision this percentage was 39% while for 1-stage revision 56%, for major partial 58% and for minor partial 85% (Table 2). Accordingly, it appears to be more difficult to eradicate the infection with a single-stage procedure.

Uncemented implants have been recommended for use in revision of infected THAs (Fink 2009, Winkler 2009). We were unable, however, to find any difference in the results for revisions performed with uncemented implants or with implants where antibiotic-loaded cements were used, either for the whole period or for 2002–2009.

Microorganisms growing in biofilms on the implant are of vital importance in the pathogenesis of prosthetic joint infection (Trampuz and Zimmerli 2006). These biofilms render the infection both difficult to diagnose and difficult to eradicate (Moojen et al. 2010). Recommendations for partial exchange revision of infected THAs stress the importance of a short duration of signs or symptoms of infection (less than 3 weeks) after the onset of infection (Zimmerli et al. 2004). Although the interval in our analyses from primary THA to the first revision was not identical with the interval from symptoms of infection until revision, most postoperative joint infections are probably the result of peroperative contamination. In our analyses, the mean time interval between the primary surgery and the revision for infection was shorter for minor partial exchange (0.7 years) than for the other procedures (2.9–3.1 years). Debridement with implant retention procedures gave good results. When we divided the interval between the primary operation and the revision procedure into 3 groups (< 3 weeks, 3–12 weeks, and > 12 weeks, we were unable to detect any influence of the time interval on the results of the revision—either for the whole group or when stratified for the different types of surgical procedures. Biofilms probably become established after a few hours, and in that respect also, 3 weeks may be much too long an interval. In accordance with this is the finding from the Mayo Clinic that duration of symptoms prior to debridement of more than 7 days is a risk factor that is independently associated with treatment failure (Marculescu et al. 2006).

In conclusion, the data in the Norwegian Arthroplasty Register showed that the 2-stage exchange procedure gave the highest survival rates after infected total hip arthroplasty. Debridement with implant retention procedures had a survival of 76% at 2 years, indicating that this is an attractive option in selected patients. Exchange of stem or cup alone gave poor results and cannot be recommended in the attempt to eradicate THA infection.
